# Changes in Short-term, Long-term, and Preventive Care Delivery in US Office-Based and Telemedicine Visits During the COVID-19 Pandemic

**DOI:** 10.1001/jamahealthforum.2021.1529

**Published:** 2021-07-09

**Authors:** Cecilia Cortez, Omar Mansour, Dima M. Qato, Randall S. Stafford, G. Caleb Alexander

**Affiliations:** 1 Johns Hopkins University, Baltimore, Maryland; 2 Monument Analytics, Baltimore, Maryland; 3 Titus Family Department of Clinical Pharmacy, School of Pharmacy, University of Southern California, Los Angeles; 4 Schaeffer Center for Health Policy and Economics, University of Southern California, Los Angeles; 5 Stanford Prevention Research Center, Stanford University School of Medicine, Stanford, California; 6 Center for Drug Safety and Effectiveness, Johns Hopkins Bloomberg School of Public Health, Baltimore, Maryland; 7 Department of Epidemiology, Johns Hopkins Bloomberg School of Public Health, Baltimore, Maryland; 8 Division of General Internal Medicine, Johns Hopkins Medicine, Baltimore, Maryland

## Abstract

**Importance:**

While the COVID-19 pandemic has been associated with some substitution of telemedicine for office-based care in the US, to our knowledge, little is known regarding the pandemic’s association with the clinical content of ambulatory care.

**Objective:**

To characterize changes in the clinical content of ambulatory care among office-based vs telemedicine encounters in the US before vs during the COVID-19 pandemic.

**Design, Settings, and Participants:**

This analysis of serial cross-sectional data from the IQVIA National Disease and Therapeutic Index was a 2-stage, stratified nationally representative audit of outpatient care in the US from January 1, 2018, through December 31, 2020. The National Disease and Therapeutic Index generates approximately 33 617 quarterly visits that are projected to 306.7 million national visits based on the survey design.

**Main Outcomes and Measures:**

(1) Prevalence of common diagnoses and (2) mix of long-term, short-term, and preventive care.

**Results:**

The mean (SD) number of projected quarterly, in-person, office-based visits was 282.1 (1.4) million in 2018 and 284.7 (10.3) in 2019 before declining to 250.8 million in quarter 1 of 2020 and 147.8 million in quarter 2 of 2020 and then increasing moderately to 181.5 million in quarter 3 of 2020 and 180.2 million in quarter 4 of 2020. The mean (SD) number of telemedicine visits was 2.8 (0.4) million in 2018 and 3.0 (0.1) million in 2019 before increasing to 8.6 million in quarter 1 of 2020 and 72.2 million in quarter 2 of 2020 and then declining notably to 43.8 million in quarter 3 of 2020 and 44.2 million in quarter 4 of 2020. Office-based care during the second through fourth quarters of 2020 involved 58.0% long-term, 23.0% short-term, and 25.6% preventive care. In contrast to office-based care, 4 of the top 10 diagnoses that were treated by telemedicine during 2020 were for psychiatric or behavioral conditions: depression, attention deficit/hyperactivity, anxiety, and bipolar disorders. Throughout this period, approximately half of office-based visits and nearly two-thirds of telemedicine visits were for established rather than new patients.

**Conclusions and Relevance:**

This cross-sectional study’s findings suggest that while telemedicine rapidly increased early during course of the COVID-19 pandemic, its use declined modestly since then. In contrast to office-based care, telemedicine was more commonly used for established patients and substantially greater delivery of psychiatric or behavioral treatments rather than preventive care.

## Key Points

**Question:**

Has the COVID-19 pandemic been associated with any changes in the clinical content of ambulatory care in the US?

**Findings:**

In this cross-sectional study of serial data from the IQVIA National Disease and Therapeutic Index, there was a moderate rebound in office-based care during the second half of 2020, while telemedicine accounted for 23.9% of care observed. Office-based care during the pandemic (quarters 2-4 of 2020) involved 58.0% long-term, 23.0% short-term, and 25.6% preventive diagnoses, while telemedicine care involved substantially greater long-term (77.2%), modestly greater short-term (26.8%), and almost no preventive (2.7%) diagnoses.

**Meaning:**

In contrast to office-based care, telemedicine was more commonly used for established patients and substantially greater delivery of psychiatric or behavioral treatments than preventive care.

## Teaser

This cross-sectional study of data from the IQVIA National Disease and Therapeutic Index examines changes in the clinical content of ambulatory care among office-based vs telemedicine encounters in the US during the COVID-19 pandemic.

## Introduction

Since the first cases were announced in the US in January 2020, the COVID-19 pandemic has caused profound disruption in the health care sector. This includes large increases in individuals without insurance, financial losses among hospitals and office practices, and delays in nonessential care.^1,2^ In addition, because of concerns regarding the potential nosocomial transmission of coronavirus infection and reorientation of the short-term care delivery system toward treating individuals with COVID-19, the pandemic has shifted the delivery of ambulatory care toward telemedicine.^3^

Analyses of administrative health care data suggest that large decreases in the provision of face-to-face care in the US occurred during the second, third, and fourth quarters of 2020 and were partially offset by increases in the use of telemedicine.^4^ Such increases have been associated with reductions in the assessment of common cardiovascular risk factors, such as blood pressure and cholesterol levels.^5^ For specific psychiatric or behavioral conditions, the total number of visits (in-person and telemedicine) increased above previous baseline levels during and before 2020.^6^

Past studies leave several questions unanswered, especially changes in the clinical content of ambulatory care. For example, it is unclear whether the conditions and diagnoses that are typically managed through face-to-face care are fundamentally different than those that have been evaluated and treated using telemedicine. Similarly, ambulatory primary care is designed to address a combination of short-term, long-term, and preventive needs, and it is unknown whether, and how, the shift to telemedicine has substantially altered this balance. We used a nationally representative audit of outpatient care to quantify changes in these components of ambulatory care among office-based vs telemedicine encounters in the US.

## Methods

### Data

We used IQVIA’s National Disease and Therapeutic Index (NDTI) to conduct a serial cross-sectional study from the first quarter of 2018 through the last quarter of 2020. The NDTI is a proprietary, 2-stage, stratified, nationally representative audit of outpatient care in the US.^7^ Investigations comparing NDTI with the National Ambulatory Medical Care Survey, a nationally representative survey sponsored by the National Center for Health Care Statistics, suggest substantively comparable estimates of ambulatory practice.^8,9,10^ The NDTI collects information from approximately 4800 clinicians each month, with participants completing a form for 2 consecutive days regarding each patient visit, including diagnoses, treatments, and demographic information. Approximately 34 000 quarterly visits are then projected to national levels after accounting for the 2-stage stratified design (306.7 million visits). The audit is randomly assigned to cover all workdays, including weekends for participants who work on those days. Clinician specialty is based on 1 of 28 specialty codes that are derived from the American Medical Association masterfile.^11^ The NDTI is limited to the contiguous US, excluding clinicians from Alaska and Hawaii, because of design considerations. We focused on office-based and telemedicine visits, for which telemedicine visits included encounters using telephones as well as web-based platforms; we excluded the fewer than 5% of face-to-face visits that took place in other settings, such as residences, nursing facilities, schools or universities, or other institutions. Our analysis, which followed the Strengthening the Reporting of Observational Studies in Epidemiology (STROBE) reporting guidelines for cross-sectional studies,^12^ was not considered human participants research and thus was exempted from further review by the Johns Hopkins Bloomberg School of Public Health institutional review board.

### Outcomes

We examined 2 outcomes. First, we characterized the most common conditions or diagnoses separately for office-based and telemedicine care. To do so, we used diagnostic information captured by NDTI at the visit level using a system similar to the *International Classification of Diseases, Ninth Revision (ICD-9)*. Two practicing clinicians (G.C.A. and R.S.S.) reviewed lists of the 100 most common diagnoses and, in some instances, combined diagnoses that were for similar conditions, such as urinary tract infections and acute cystitis or types 1 and 2 diabetes.

Second, we classified each visit into 1 of 3 mutually exclusive categories based on whether care addressed a short-term, long-term, or preventive issue. We considered conditions such as those associated with respiratory infections or genitourinary infections, headaches, and musculoskeletal pain as short-term except when otherwise noted. We classified cardiovascular conditions such as diabetes and hypertension, psychiatric and behavioral disorders such as conduct disorders or anxiety, and conditions such as gastroesophageal reflux and obesity as long term. Preventive care included well-child and pre-employment examinations, contraceptive advice, and immunizations and other diagnoses that are connoted by a V-code, which is used by *ICD-9* to capture health care encounters that are not the result of a disease or injury.^13^

### Analysis

We used descriptive statistics to conduct our analysis. All estimates are weighted using IQVIA’s proprietary weight algorithm to account for the complex design of NDTI and generate national projections of ambulatory care. We also used standardized errors to estimate 95% confidence intervals around estimates of interest. Analyses were conducted using an online platform for NDTI that is provided by IQVIA and MS Excel.

First, we extracted information regarding the total number of quarterly visits over the 36-month study period and stratified these into those that took place through office-based vs telemedicine care. We examined the data for general trends, inflection points, and potentially associated outliers. Second, we stratified the data by diagnosis to establish the top 100 diagnoses. Third, we stratified the data after categorizing each diagnosis into 1 of 3 mutually exclusive categories: short-term, long-term, or preventive. Thus, an individual who was seen for diabetes and an immunization would contribute twice to this analysis: once to the long-term condition category (diabetes) and once to the preventive category (immunization). Fourth, we stratified the data into new and subsequent visits by yearly quarters. Finally, we characterized the use of office-based and telemedicine care stratified by clinician type, grouping clinicians into those practicing in primary care, specialty care, and surgical fields. We defined *primary care clinicians* as family practice, general practice, geriatrics, internal medicine, osteopathic medicine, and pediatrics and classified obstetricians-gynecologists with other surgical clinicians.

## Results

**Figure 1.**
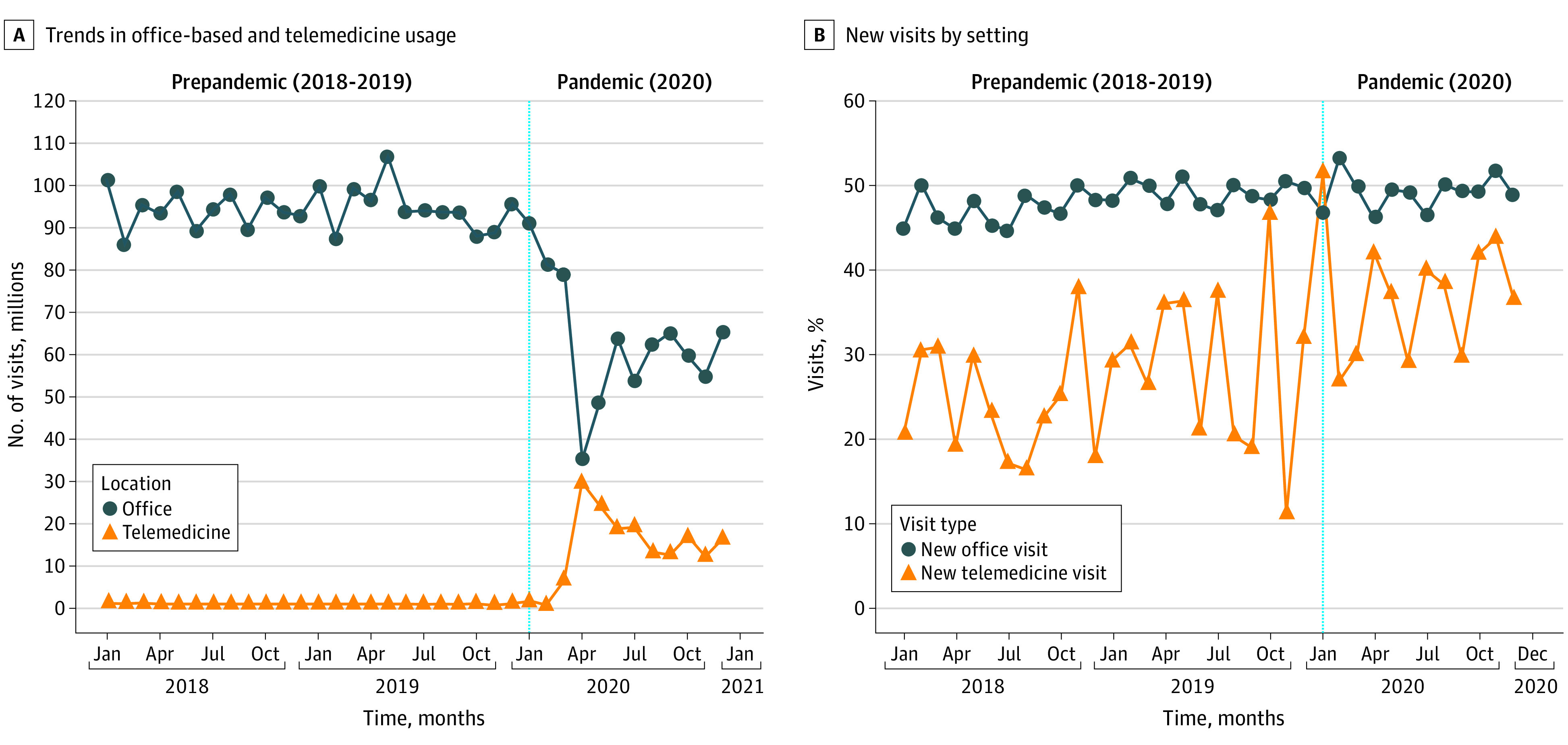
Monthly Trends in Office-Based and Telemedicine Visits and Percentage of New Visits by Setting in the US From 2018 to 2020 Based on a total of 3240 million visits. Figure generated with permission from IQVIA.

Office-based care volume was fairly stable between quarter 1 of 2018 and quarter 4 of 2019, ranging from between 101.5 million and 86.5 million visits per month, before declining slightly through quarter 1 of 2020 to a mean (SD) of 84.0 (6.5) million visits per month, and more abruptly to 49.3 (14.3) million visits in quarter 2 of 2020 before increasing modestly to 60.5 (5.8) million in quarter 3 of 2020 and 60.1 (5.3) million in quarter 4 of 2020 (Figure 1). Trends in telemedicine visits were largely reciprocal to those of office-based care, remaining plateaued through quarter 1 of 2020 before increasing markedly in quarter 2 of 2020 and declining notably in quarters 3 and 4 of 2020.

### Delivery of Short-term, Long-term, and Preventive Care

There were substantial shifts in the delivery of short-term, long-term, and preventive ambulatory care among all clinicians, including primary care clinicians and specialists, which were stratified by calendar quarter and whether the care was delivered through office-based or telemedicine encounters (Table 1). For example, based on the top 100 office-based and telemedicine diagnoses, the total number of office-based visits during the 4 quarters of 2018 and 2019 represented a mean (SD) between 128.4 (0.3) and 132.0 (1.8) million visits per quarter. Compared with these historical trends, the total number of office-based visits declined to 114.8 million during quarter 1 of 2020, representing a 10.6% reduction from mean quarter 1 of 2018/2019. There were even larger declines in officed-based visits in quarters 2 (−49.9%), 3 (−35.1%), and 4 of 2020 (−43.9%) compared with historical trends. The top 100 office-based diagnoses represented 76.0% of all office-based diagnoses in 2020, and the top 100 telemedicine diagnoses represented 83.5% of all telemedicine diagnoses in 2020.

**Table 1.** Short-term, Long-term, and Preventive Diagnoses During a Total of 1.49 Million Office-Based and Telemedicine Visits Across All Specialties in the US From 2018 to 2020^a^

Care	Quarter 1			Quarter 2			Quarter 3			Quarter 4		
	2018/2019 (mean) visits, No. (range)	2020 (mean) Visits, No. (range)	Change, %	2018/2019 (mean) Visits, No.	2020 (mean) Visits, No. (range)	Change, %	2018/2019 (mean) Visits, No. (range)	2020 (mean) Visits, No. (range)	Change, %^b^	2018/2019 (mean) Visits, No. (range)	2020 (mean) Visits, No. (range)	Change, %
**Office-based care**												
Total visits	128.4 (122.8 to 133.9)	114.8 (109.7 to 120.0)	−10.6	131.7 (126.0 to 137.4)	66.0 (62.5 to 69.5)	−49.9	132.0 (126.3 to 137.7)	85.7 (81.6 to 89.8)	−35.1	128.7 (123.1 to 134.3)	72.2 (68.4 to 75.6)	−43.9
Short term	30.2 (27.8 to 32.6)	28.2 (25.8 to 30.5)	−6.7	29.9 (28.0 to 31.8)	14.2 (12.8 to 15.6)	−52.6	27.3 (25.1 to 29.4)	17.7 (15.9 to 19.4)	−35.2	28.6 (26.3 to 30.8)	19.6 (17.7 to 21.6)	−31.3
Long term	82.2 (78.4 to 85.9)	73.6 (69.8 to 77.3)	−11.3	84.7 (80.8 to 88.5)	39.5 (36.7 to 42.3)	−53.4	85.5 (81.5 to 89.4)	51.5 (48.5 to 54.5)	−53.8	83.5 (79.7 to 87.4)	38.9 (26.2 to 41.7)	−53.5
Preventive	27.1 (25.0 to 29.3)	24.0 (22.1 to 26.0)	−11.4	29.5 (27.2 to 31.8)	18.4 (16.5 to 20.2)	−18.6	31.6 (29.4 to 33.7)	24.0 (22.0 to 25.9)	−24.0	29.1 (29.4 to 33.7)	14.9 (13.4 to 16.4)	−48.9
**Telemedicine care**												
Total visits	1.7 (1.2 to 2.2)	4.8 (3.8 to 5.6)	+178.2	1.6 (1.2 to 2.0)	40.3 (37.8 to 42.3)	+2401.0	1.7 (1.2 to 2.1)	24.9 (22.7 to 26.9)	+1470.0	1.5 (1.1 to 1.9)	22.7 (20.9 to 24.6)	+1435.2
Short term	0.4 (0.2 to 0.5)	1.3 (0.9 to 1.6)	+232.3	0.3 (0.2 to 0.5)	9.9 (8.5 to 11.2)	+3003.1	0.4 (0.2 to 0.6)	6.1 (5.1 to 7.0)	+1370.0	0.3 (0.2 to 0.5)	7.6 (6.4 to 8.8)	+2100.5
Long term	1.3 (0.9 to 1.6)	3.6 (2.8 to 4.3)	+2455.0	1.3 (0.9 to 1.6)	32.9 (30.6 to 35.2)	+2504.5	1.1 (0.8 to 1.4)	20.2 (18.5 to 21.8)	+1711.8	1.1 (0.8 to 1.4)	14.8 (13.3 to 16.3)	+1227.3
Preventive	0.1 (0.05 to 0.15)	0.02 (0.01 to 0.03)	−81.8	0.08 (0.04 to 0.1)	1.1 (0.8 to 1.4)	+1277.3	0.08 (0.04 to 0. 1)	0.8 (527 to 1117)	+945.0	0.1 (45 to 151)	0.5 (0.3 to 0.7)	+811.0
**All care**												
Total visits	130.1 (124.4 to 135.7)	119.6 (114.2 to 124.9)	−8.1	133.3 (127.5 to 139.1)	106.4 (101.6 to 111.1)	−20.2	133.6 (127.8 to 139.3)	110.6 (105.6 to 115.5)	−17.2	130.2 (124.5 to 135.8)	94.9 (90.6 to 99.2)	−27.1
Short term	30.6 (28.5 to 32.6)	29.4 (27.0 to 31.8)	−3.7	30.2 (28.2 to 32.3)	24.1 (22.1 to 26.0)	−20.4	27.7 (25.5 to 29.8)	23.7 (21.8 to 25.6)	−14.3	28.9 (26.6 to 31.2)	27.1 (24.9 to 29.3)	−6.7
Long term	84.2 (80.4 to 88.0)	77.1 (73.2 to 81.0)	−8.4	85.9 (82.0 to 89.9)	72.4 (68.7 to 76.0)	−15.8	86.6 (82.6 to 90.5)	71.7 (68.1 to 75.3)	−17.2	84.7 (80.8 to 88.5)	53.6 (50.5 to 56.7)	−36.6
Preventive	27.2 (25.1 to 29.4)	28.1 (25.8 to 30.4)	+3.3	29.6 (27.2 to 31.9)	19.5 (17.5 to 21.4)	−34.2	31.6 (29.5 to 33.8)	24.8 (22.8 to 29.8)	−21.6	29.1 (26.8 to 31.4)	15.4 (13.9 to 16.9)	−47.3

^a^ Estimates are based on a combination of the top 100 office-based and telemedicine diagnoses. Numbers in parentheses represent 95% CIs. Source: IQVIA National Disease and Therapeutic Index, 2018 to 2020.

^b^ Percentage change calculated as difference between a given quarter and the mean visit volume for that same quarter in 2018 and 2019.

In addition to depicting large increases in the use of telemedicine during quarters 1 (178.2%), 2 (2401%), 3 (1470%), and 4 of 2020 (1435%), Table 1 also depicts how changes in short-term, long-term, and preventive care occurred across these different modalities. For example, between quarters 2 and 4 of 2020, declines in preventive care (−21.6% to −47.3%) were greater than those for long-term (−15.8% to −36.6%) or short-term (−6.7% to −20.4%) care when examining office-based and telemedicine visits.

**Figure 2.**
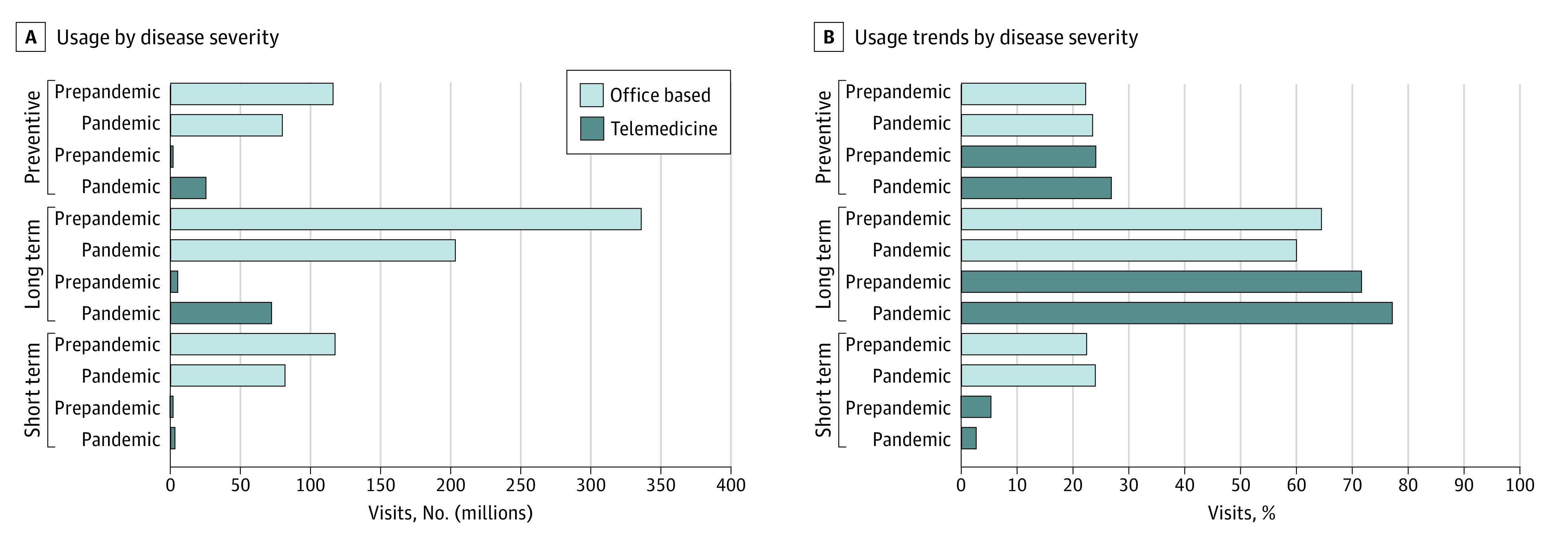
Trends in Short-term, Long-term, and Preventive Visits Before (2018/2019) and During (2020) the COVID-19 Pandemic by Visit Setting Based on a total of 1485 million visits. Figure generated with permission from IQVIA.

Figure 2 provides additional information on the delivery of short-term, long-term, and preventive care during the COVID-19 pandemic in 2020, including the number of office visits (panel A) and proportion of visits accounted for by different types of care (panel B). During 2020, short-term care had an increase in the proportion of visits compared with its 2018/2019 mean in office-based (23.5% in 2020 vs 22.2% in 2018/2019) and telemedicine visits (26.8% in 2020 vs 24.0% in 2018/2019). Long-term care experienced a decrease in the proportion of visits for office-based visits (60% in 2020 vs 64.5% in 2018/2019) but an increase for telemedicine visits (77.1% in 2020 vs 71.6% in 2018/2019). Preventive care had an increase in the proportion of office-based visits (24.0% in 2020 vs 22.5% in 2018/2019) but a decrease in telemedicine visits during the same period (2.6% in 2020 vs 5.3% in 2018/2019).

eTable 4 in the Supplement presents the use of all office-based and telemedicine visits stratified by clinician type. For example, the total number of primary care visits from 2018 and 2019 was a mean (SD) between 137.1 (2.5) million and 140.6 (7.1) million. Compared with historical trends, the total number of primary care visits declined to 129.7 million during quarter 1 of 2020, representing a 7.2% reduction from mean quarter 1 of 2018/2019. There were even larger reductions in quarters 2 (−22.3%), 3 (−20.8%), and 4 of 2020 (−16.6%). Specialty care decreased between −9.4% and −21.8% during the same period. Surgical care decreased the most by −24.7% and −38.1% (quarters 2-4 in 2020). Despite the increase in telemedicine (between 1470% and 2400% during quarters 2-4 in 2020), there was an overall loss of care among all specialties.

### Trends in Treating Specific Diagnoses

**Figure 3.**
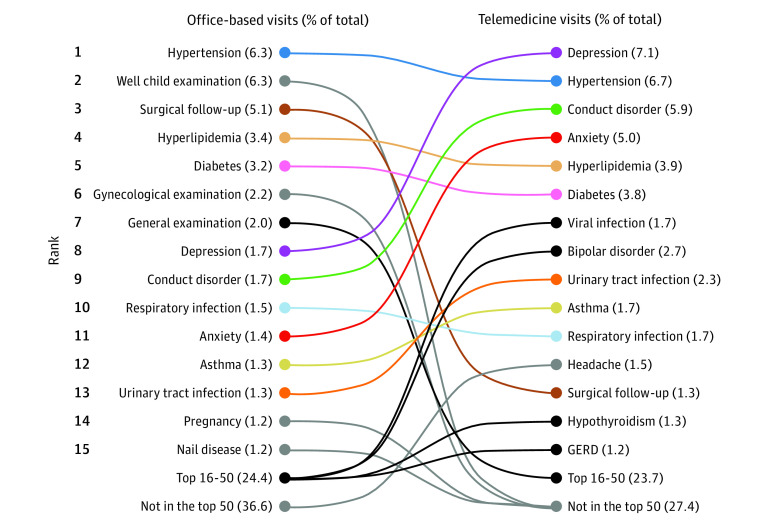
Trends in Top Office-Based and Telemedicine Diagnoses Across All Visits During 2020 Based on a total of 3228 million visits. Figure generated with permission from IQVIA.

Figure 3 depicts the most common diagnoses that were managed during office-based and telemedicine care across all clinician types during 2020. Of these, 9 diagnoses were included on both lists of the 15 most common conditions, whereas each list contained 6 diagnoses that were unique. Psychiatric and behavioral conditions, such as anxiety, depression, and conduct disorder, were represented in only 3 of the top 15 office-based diagnoses compared with 4 of the top 15 telemedicine diagnoses. Preventive care accounted for 4 of the top 15 office-based diagnoses and 2 of the top 15 telemedicine diagnoses. Viral infection ranked seventh of the top 15 telemedicine diagnoses, representing approximately 2% of the total telemedicine visits that were examined. For office-based medicine, viral infection was ranked in the top 50 visits, but not in the top 15, and accounted for 0.5% of visits.

eTable 1 in the Supplement provides additional information regarding the most common diagnoses that were addressed during office-based and telemedicine care across all clinician types, including rankings of the top 25 diagnoses managed in each setting, as well as total visit counts. The findings of eTable 1 in the Supplement are similar to those depicted in Figure 3, including greater psychiatric and behavioral care and less preventive care delivered through telemedicine than office-based encounters during the final 3 calendar quarters of 2020.

### Trends in Primary Care, Specialty, and Surgical Visits

eTable 2 in the Supplement presents the use of all office-based and telemedicine visits as stratified by clinician type. For example, specialty care saw a decrease in office-based visits ranging from −41.2% to −55.7% during quarters 2 to 4 of 2020 compared with the mean visits for 2018/2019. Telemedicine specialty care increased between 2136% and 3364% during the same period. Despite the increase in telemedicine, there was an overall decrease in specialty care that ranged from −9.4% to −21.8%. Primary care followed a similar pattern, with a decrease in office-based visits (−33.7%, −50.0%), an increase in telemedicine (+1417%, +2250%), and an overall decrease (−16.6%, −22.3%). Surgical care followed the same trend of decreased office-based visits (−28.8%, −41.7%), an increase in telemedicine visits (+700%, +1485%), and an overall decrease (−11.4%, −38.1%).

eTable 3 in the Supplement further characterizes the volume of care provided by primary care, specialty, and surgical clinicians before and during the pandemic as stratified by site of care. For example, in 2020, primary care was the most common clinician for office-based care, accounting for 48.8% of visits, followed by obstetricians-gynecologists (8.7% of visits) and podiatry (4.9%). For telemedicine, primary care was also the most common clinician during 2020, accounting for 53.9% of visits, followed by psychiatry (16.9%) and cardiology (4.6%).

Table 2 provides the proportion of all visits for a given clinician type that were accounted for by telemedicine care, highlighting the 5 fields with the lowest and highest percentage of telemedicine visits during 2020, as well as their historical use of telemedicine. For example, of the 28 specialty categories included in NDTI, those with the greatest proportion of all of their visits delivered by telemedicine during 2020 were psychiatry (61.7%), gastroenterology (35.1%), neurology (31.3%), cardiology (26.1%), and primary care (19.9%). By contrast, those with the fewest visits accounted for by telemedicine included general surgery (5.3% of all general surgery visits), other surgery (4.6%), orthopedic surgery (3.4%), ophthalmology (2.2%), and podiatry (1.7%).

**Table 2.** Highest and Lowest Percentage of 933 Million Telemedicine Visits Across Specialties From 2018 to 2020^a^

Rank	Specialty	No. of quarterly visits in thousands (% of telemedicine visits)	
		(2018/2019)	(2020)
First	Psychiatry	482.5 (3.6)	7259.1 (61.7)
Second	Gastroenterology	40.1 (0.8)	1525.9 (35.1)
Third	Neurology	65.1 (1.1)	1700.0 (31.3)
Fourth	Cardiology	38.6 (0.4)	1979.1 (26.1)
Fifth	Primary care	1159.0 (0.5)	23 125.2 (19.9)
24th	General surgery	8.7 (0.1)	267.3 (5.3)
25th	All other surgery	40.0 (0.3)	295.8 (4.6)
26th	Orthopedic surgery	36.0 (0.3)	295.9 (3.4)
27th	Ophthalmology	23.4 (0.2)	193.4 (2.2)
28th	Podiatry	15.0 (0.13)	156.3 (1.7)
All visits		23 033.0 (1.0)	42 925.2 (18.4)

^a^ Top-ranking specialties were limited to major specialties with at least 3% of the volume of telemedicine visits. Source: IQVIA National Disease and Therapeutic Index, 2018 to 2019.

### Trends in Establishment of Care

eTable 4 in the Supplement depicts trends in new vs subsequent visits in office-based and telemedicine. For example, office-based visits accounted for 281.8 million visits in mean quarter 3 of 2018/2019, with 47.8% being new visits and 53.1% being subsequent visits. Although the number of office-based visits decreased by 35.6% (181.5 million visits) in quarter 3 of 2020, the proportion of new and subsequent office-based visits stayed steady with 48.9% new visits and 52.3% subsequent visits. In contrast, 36.7% of telemedicine visits were for new patients in quarter 3 of 2020.

Figure 1 provides monthly time trends in the use of office-based and telemedicine care for new visits. The proportion of telemedicine visits accounted for by new care ranged between a monthly mean (SD) of 18.8% (3.3%) to 31.6% (17.9%) from quarter 1 of 2018 through quarter 4 of 2019, increasing to a monthly mean (SD) of 37.1% (6.5%; 2020, quarter 2), 36.7% (5.6%; 2020, quarter 3) and 40.5% (3.8%; 2020, quarter 4) since the COVID-19 pandemic began.

## Discussion

While the COVID-19 pandemic has driven increases in the use of telemedicine, much less is known about how the pandemic has changed the clinical content of ambulatory care. We used an ongoing, nationally representative office-based physician audit to examine the prevalence of common diagnoses and the mix of long-term, short-term, and preventive care delivery in the US. There was a moderate rebound in office-based care and decrease in telemedicine during the second half of 2020. Nevertheless, even in quarter 4 of 2020, telemedicine accounted for 20.1% of care observed, far more than the historical levels (2018/2019 mean) of 1.0%. In contrast to office-based care, telemedicine was more commonly used for established patients and substantially greater delivery of psychiatric or behavioral treatments rather than preventive care. These findings are important because of how little is known regarding the pandemic’s association with care delivery.

Our finding that telemedicine is being used often for psychiatric and behavioral conditions is noteworthy and is likely associated with increased demand for such services as well as the ability for their delivery through telephone- and web-based platforms. Pandemic-related increases in mental health conditions, such as depressive disorder, anxiety disorder, and suicidal ideation, among adults within the US,^14^ as well as elsewhere, have been noted.^15^ Despite technology barriers, using telemedicine for psychiatry allows for more patient flexibility on the location of delivery of care, reduces the need for transportation, and allows patients to attend appointments without taking time off of work.^16^ As before the pandemic, much of the care delivery for psychiatric and behavioral conditions, such as anxiety, depression, conduct disorder, and bipolar disorder, continues to be provided by primary care physicians.^17,18^

We also found markedly less preventive care being delivered by telemedicine than office-based care, including immunizations, well child examinations, routine histories and physical examinations, and counseling or other patient engagement, that is not the result of a disease or injury. Such findings extend prior reports regarding potential pandemic-related declines in cancer screening services such as mammography^19^ and colorectal cancer prevention.^20,21^ Decreases in some types of preventive services, such as immunizations and well child examinations, are obvious, given that these do not lend themselves to delivery by telemedicine. Less intuitively, other types of preventive care, such as preventive counseling regarding matters such as anticipatory child development, diet, exercise, or other behavioral dimensions of care, may be crowded out by short-term and long-term care needs that are competing for the attention of clinicians and patients during telemedicine visits. Our findings regarding shifts in long-term, short-term, and preventive care may be associated with reimbursement requirements, because some care, such as pediatric well visits and visits for non-Medicare beneficiaries, must still be performed in person to be reimbursed.

Substantial uncertainty remains regarding the degree to which the COVID-19 pandemic will fundamentally transform the role of telemedicine care delivery in the US. On one hand, many historical barriers to telemedicine adoption, which range from interstate licensure requirements to clinician and patient ambivalence,^22,23^ are likely to persist in the postpandemic era. In addition, coverage and reimbursement policies have also hindered the historic adoption of telemedicine, and while federal and state policy makers modified these during the pandemic, it is unclear whether they will be permanently used once public health emergency provisions are no longer in place. The COVID-19 pandemic has galvanized many clinical practices to invest in technologies and redesign workflows to better accommodate telemedicine delivery.^24^ Patients and clinicians may have greater comfort with and acceptance of telemedicine platforms because of their successful use during the pandemic.

Interestingly, despite the large decrease in office-based visits and increased telemedicine delivery early during the COVID-19 pandemic, we noted a rebound in in-person visits during the third and fourth quarters of 2020. This rebound may have been associated with a relaxation of clinical policies that were previously restricting such care, pent-up demand on the part of patients, and greater comfort on the part of clinicians and patients in managing face-to-face workflows safely.

### Limitations

Our study has several limitations. First, our definition of telemedicine includes care delivered by telephone and through web-based video-conferencing platforms, yet the technologies, user experience, and ultimate clinical outcomes that are associated with these may differ. Second, our data do not allow for us to quantify quality of care. Third, although we examined data through 2020, the COVID-19 pandemic remains a dynamic, rapidly changing phenomenon; ongoing surveillance is needed to determine the future evolution of the associations that we describe. Fourth, our study design does not allow for us to understand how individuals are triaging between office-based and telemedicine care, yet this remains an important question for clinicians and policy makers.

## Conclusions

In this cross-sectional study, telemedicine use increased substantially during the early phase of the pandemic, then decreased slightly, and steadied at a visit rate much larger than before. Despite these increases, diagnosis of short-term, long-term, and preventive, as well as visits by all physician specialties, decreased. When diagnosing, telemedicine was used more frequently for patients with psychiatric disorders, while office-based care was used more frequently for preventive visits. Primary care clinicians delivered the most care for office-based and telemedicine visits despite the difference in diagnosis between the 2 visit types, and has been associated with a change in the structure and clinical content of ambulatory care.

## Notes

Supplement. **eTable 1.** Most Common Office-based and Telemedicine Diagnoses Across All Specialties, 2018-2020 (N=3,228 million). **eTable 2.** Trends in Office-based and Telemedicine Visits by Care Type, 2018-2020 (in thousands, N=3,219 million). **eTable 3.** Most Common Office-Based and Telemedicine Visits Across Specialties, 2018-2020 (N=3,228 million). **eTable 4.** Percentage of New and Subsequent Visits Among Office-Based and Telemedicine Visits, 2018-2020 (N=3,240 million).
