# Supplementary material for: Changes in Short-term, Long-term, and Preventive Care Delivery in US Office-Based and Telemedicine Visits During the COVID-19 Pandemic
Source: JAMA Health Forum. 2021 Jul 9;2(7):e211529. doi: 10.1001/jamahealthforum.2021.1529 (PMC8796900; doi:10.1001/jamahealthforum.2021.1529)
Supplement: Supplement. — eTable 1. Most Common Office-based and Telemedicine Diagnoses Across All Specialties, 2018-2020 (N=3,228 million). eTable 2. Trends in Office-based and Telemedicine Visits by Care Type, 2018-2020 (in thousands, N=3,219 million). eTable 3. Most Common Office-Based and Telemedicine Visits Across Specialties, 2018-2020 (N=3,228 million). eTable 4. Percentage of New and Subsequent Visits Among Office-Based and Telemedicine Visits, 2018-2020 (N=3,240 million). [file jamahealthforum-e211529-s001.pdf]

Supplemental Online Content

Cortez C, Mansour O, Qato DM, Stafford RS, Alexander GC. Changes in short-term, long-term, and preventive care delivery in US office-based and telemedicine visits during the COVID-19 pandemic. *JAMA Health Forum*. 2021;2(7):e211529. doi:10.1001/jamahealthforum.2021.1529

eTable 1. Most Common Office-based and Telemedicine Diagnoses Across All Specialties, 2018-2020 (N=3,228 million)

eTable 2. Trends in Office-based and Telemedicine Visits by Care Type, 2018-2020 (in thousands, N=3,219 million)

eTable 3. Most Common Office-Based and Telemedicine Visits Across Specialties, 2018-2020 (N=3,228 million)

eTable 4. Percentage of New and Subsequent Visits Among Office-Based and Telemedicine Visits, 2018-2020 (N=3,240 million).

This supplemental material has been provided by the authors to give readers additional information about their work.

© 2021 Cortez C et al. *JAMA Health Forum.*

eTable 1. Most Common Office-based and Telemedicine Diagnoses Across All Specialties, 2018-2020 (N=3,228 million).

	**Pre-Pandemic (2018/2019)**		**Pandemic (2020)**	
**Rank**	**Office Visits (% of total)**	**Telemedicine Visits (% of total)**	**Office Visits (% of total)**	**Telemedicine Visits (% of total)**
1	Hypertension (6.5%)	Depression (8.6%)	Hypertension (6.3%)	Depression (7.1%)
2	Surgical Follow Up (5.0%)	Conduct Disorder (7.6%)	Well Child Exam (5.3%)	Hypertension (6.7%)
3	Well Child Exam (4.2%)	Anxiety (5.0%)	Surgical Follow Up (5.1%)	Conduct Disorder (5.9%)
4	Hyperlipidemia (3.4%)	Hypertension (4.9%)	Hyperlipidemia (3.4%)	Anxiety (5.0%)
5	Diabetes (3.1%)	Contraceptive Management	Diabetes (3.2%)	Hyperlipidemia (3.9%)
6	Depression (2.5%)	Hyperlipidemia (3.4%)	Gynecological Exam (2.2%)	Diabetes (3.8%)
7	Conduct Disorder (2.0%)	UTI (3.3%)	General Exam (2.0%)	Viral Infection (1.7%)
8	Gynecological Exam (2.0%)	Respiratory Infection (2.8%)	Depression (1.7%)	Bipolar Affective Disorder (2.7%)
9	Respiratory Infection (2.0%)	Vaginal Symptoms (2.3%)	Conduct Disorder (1.7%)	UTI (2.3%)
10	General Exam (1.9%)	Sleep Disorder (2.0%)	Respiratory Infection (1.5%)	Asthma (1.7%)
11	Anxiety (1.63%)	Hypothyroidism (1.9%)	Anxiety (1.4%)	Respiratory Infection (1.7%)
12	Asthma (1.3%)	Surgical Follow Up (1.7%)	Asthma (1.3%)	Headache (1.5%)
13	UTI (1.3%)	Bipolar Affective Disorder (1.6%)	UTI (1.3%)	Surgical Follow Up (1.3%)
14	Pregnancy (1.2%)	Diabetes (1.5%)	Pregnancy (1.2%)	Hypothyroidism (1.3%)
15	Pharyngitis (1.1%)	Asthma (1.5%)	Nail Disease (1.2%)	GERD (1.2%)
16	Hypothyroidism (1.1%)	Rhinitis (1.3%)	Hypothyroidism (1.1%)	Rhinitis (1.2%)
17	Rhinitis (1.1%)	Adjustment Disorder (1.0%)	Rhinitis (1.0%)	Sleep Disorder (1.2%)
18	Cataracts (1.0%)	GERD (1.0%)	GERD (1.0%)	Atrial Fibrillation/Flutter (1.2%)
19	GERD (1.0%)	Influenza (0.8%)	Glaucoma (1.0%)	Dermatitis (1.1%)
20	Nail Disease (1.0%)	Headache (0.8%)	Vaginal Symptoms (1.0%)	Vaginal Symptoms (1.0%)
21	Vaginal Symptoms (0.9%)	Atrial Fibrillation/Flutter (0.8%)	Dermatitis (1.0%)	Adjustment Disorder (1.0%)
22	Glaucoma (0.9%)	Acute Sinusitis (0.8%)	Osteoarthritis (0.9%)	Acute sinusitis (0.9%)
23	Atrial Fibrillation/Flutter (0.9%)	Pharyngitis (0.8%)	Lower Back Pain (9.4%)	COPD (0.9%)
24	Otitis Media (0.9%)	General Exam (0.8%)	Cataracts (0.9%)	Lower Back Pain (0.8%)
25	Headache (0.9%)	Menopausal Symptoms (0.6%)	Atrial Fibrillation/Flutter (8.4%)	OCD (0.8%)
	All other (51.5%)	All other (39.3%)	All other (51.2%)	All Other (41.0%)
All Visits	2,271.6 (2,173.3-2,370.0) (in millions)	23.0 (21.2, 24.8) (in millions)	761.6 (727.4-795.8) (in millions)	171.7 (164.0-179.4) (in millions)

UTI urinary tract infection; **GERD** gastroesophageal reflux disease; **COPD** chronic obstructive pulmonary disease; **OCD** obsessive compulsive disorder.
Source: IQVIA National Disease and Therapeutic Index, 2018-2020.

© 2021 Cortez C et al. *JAMA Health Forum.*

eTable 2. Trends in Office-based and Telemedicine Visits by Care Type, 2018-2020 (in thousands, N=3,219 million).

	Quarter 1			Quarter 2			Quarter 3			Quarter 4		
	2018/2019 Average Visits, n	2020 Visits, n	% Change	2018/2019 Average Visits, n	2020 Visits, n	% Change	2018/2019 Average Visits, n	2020 Visits, n	% Change*	2018/2019 Average Visits, n	2020 Visits, n	% Change
**Office-Based Care**												
Total Visits	284.4 (272.1-	250.8 (239.6-	-11.8	289.0 (276.5-301.5)	147.8 (141.2-154.5)	-48.8	281.8 (269.6-	181.5 (173.4-	-35.6	278.4 (266.3-	180.2 (172.1-	-35.3
Type of Care												
Primary	138.1 (132.1-	124.0 (118.4-	-10.2	138.9 (132.9-144.9)	69.4 (65.7-73.2)	-50.0	138.1 (132.1-	88.1 (83.9, 92.2)	-36.2	135.5 (129.6-	89.8 (85.5-	-33.7
Specialty	74.2 (70.6-77.8)	63.1 (59.6-66.5)	-15.0	76.2 (72.5-79.8)	33.7 (31.3-36.1)	-55.7	71.6 (68.2-75.1)	42.1 (39.4-44.8)	-41.2	72.1 (68.6-75.6)	49.1 (46.0-52.2)	-42.8
Surgical	72.2 (68.7-75.7)	63.8 (60.4-67.2)	-11.6	73.9 (70.3, 77.5)	44.7 (41.8-47.5)	-39.5	72,096 (68.6-75.9)	51.3 (48.4-54.3)	-28.8	70.7 (67.3-74.1)	41.3 (38.7-43.9)	-41.7
**Telemedicine Care**												
Total Visits	3.1 (2.5-3.7)	8.6 (7.4-9.9)	+177.8	2.8 (2.2-3.5)	72.2 (68.5-75.8)	+2400	2.7 (2.1-3.3)	43.8 (41.0-46.6)	+1510	2.8 (2.2-3.5)	44.2 (41.4-	+1470.1
Type of Care												
Primary Care	1.6 (1.2-2.1)	5.7 (4.7-6.7)	+246.9	1.7 (1.2-2.2)	39.8 (37.0-42.6)	+2250	1.5 (1.1-1.9)	22.4 (20.6-24.2)	+1417	1.6 (1.1-2.0)	24.6 (22.6-26.6)	+1485.8
Specialty Care	0.9 (0.6-1.2)	2.3 (1.7-2.8)	+157.1	0.8 (0.5-1.0)	26.4 (24.2-28.6)	+3364	0.8 (0.5-1.1)	18.1 (16.3-19.9)	+2200	0.8 (0.5-1.0)	16.9 (15.2-18.6)	+2136.8
Surgical Care	0.6 (0.4-0.8)	0.7 (0.4-0.9)	+16.6	0.4 (0.2-0.5)	5.9 (4.9-7.0)	+1485	0.4 (0.2-0.6)	3.3 (2.8-4.3)	+700	0.4 (0.3, 0.6)	2.8 (2.1-3.5)	+528.43
**All Care**												
Total Visits	287.5 (275.1-	259.5 (247.8-	-9.8	291.8 (279.1-304.4)	220.0 (210.1-229.7)	-24.6	284.5 (272.2-	225.3 (215.2-	-20.8	281.1 (268.9-	224.5 (214.4-	-20.2
Type of Care												
Primary Care	139.7 (133.7-	129.7 (123.9-	-7.2	140.7 (134.5-146.7)	109.3 (104.4-114.2)	-22.3	139.6 (133.5-	110.5 (105.5-	-20.8	137.1 (131.1-	114.4 (109.3,	-16.6
Specialty Care	75.0 (71.4-78.6)	65.3 (61.8-68.8)	-12.9	76.9 (73.2-80.6)	60.1 (56.9-63.3)	-21.8	72.5 (68.9-76.0)	60.3 (57.0-63.5)	-16.8	72.9 (69.3-76.4)	66.0 (62.5-	-9.38
Surgical Care	72.8 (69.3-76.3)	64.5 (61.0-67.9)	-11.4	74.3 (70.7-77.9)	50.6 (47.7-53.5)	-31.9	72.5 (69.0-76.0)	54.6 (51.4-57.7)	-24.7	71.2 (67.7-74.6)	44.0 (41.2-	-38.1

Source: IQVIA National Disease and Therapeutic Index, 2018-2020. Numbers in parentheses represent 95% confidence intervals.

© 2021 Cortez C et al. *JAMA Health Forum.*

eTable 3. Most Common Office-Based and Telemedicine Visits Across Specialties, 2018-2020 (N=3,228 million).

	**Office-Based**		**Telemedicine**	
**Rank**	**2018/2019**	**2020**	**2018/2019**	**2020**
1	Primary Care (48.5%)	Primary Care (48.8%)	Primary Care (55.2%)	Primary Care (53.9%)
2	OB/GYN (7.9%)	OB/GYN ((8.7%)	Psychiatry (16.8%)	Psychiatry (16.9%)
3	Psychiatry (4.5%)	Podiatry (4.9%)	OB/GYN (10.3%)	Cardiology (4.6%)
4	Ophthalmology (4.4%)	Ophthalmology (4.6%)	Otolaryngology (2.4%)	Neurology (4.0%)
5	Orthopedic Surgery (4.3%)	Orthopedic Surgery (4.4%)	Neurology (2.3%)	Gastroenterology (3.6%)
6	Podiatry (4.0%)	All Other Surgery (3.2%)	Pulmonary Diseases (1.8%)	OB/GYN (3.3%)
7	Cardiology (3.1%)	Cardiology (2.9%)	Oncology (1.4%)	Emergency Medicine (1.7%)
8	Dermatology (2.9%)	Dermatology (2.9%)	Gastroenterology (1.4%)	Endocrinology (1.5%)
9	All other Surgery (2.7%)	Oncology (2.7%)	All Other Surgery (1.4%)	Nephrology (1.3%)
10	Oncology (2.5%)	General Surgery (2.5%)	Cardiology (1.3%)	Oncology (1.1%)
	All other (12.7%)	All other (14.46%)	All other (9.5%)	All other (8.2%)
All Visits	2,271.6 (2,173.3-2,370.0) (in millions)	761.6 (727.4-79.76) (in millions)	23.0 (21.2-24,843) (in millions)	171.7 (164.0-179.4) (in millions)

Values in parentheses represent proportion of all visits in a given time period accounted for by a particular specialty. Thus, of all office visits that took place during 2020, 48.8% were accounted for by primary care clinicians.
Source: IQVIA National Disease and Therapeutic Index, 2018-2020**.**

© 2021 Cortez C et al. *JAMA Health Forum.*

eTable 4. Percentage of New and Subsequent Visits Among Office-Based and Telemedicine Visits, 2018-2020 (N=3,240 million).

	Quarter 1		Quarter 2		Quarter 3		Quarter 4	
	2018/2019 Average, %	2020 Visits, %	2018/2019 Average Visits, %	2020 Visits, n	2018/2019 Average Visits, %	2020 Visits, %	2018/2019 Average Visits, %	2020 Visits, %
**Office-Based Care**								
New Visits	48.4	50.0	47.7	48.7	47.8	48.9	49.0	49.4
Subsequent Visits	52.7	51.0	53.4	52.5	53.1	52.3	52.3	50.6
**Telemedicine Care**								
New Visits	28.1	33.5	27.5	37.1	23.3	36.7	29.3	40.4
Subsequent Visits	72.1	67.0	72.4	63.5	77.7	64.0	71.1	59.6
**All Care**								
New Visits	48.1	49.5	47.7	44.8	47.6	46.4	48.0	47.6
Subsequent Visits	51.9	50.5	52.2	55.1	52.3	53.6	51.7	52.3

Source: IQVIA National Disease and Therapeutic Index, 2018-2020.

© 2021 Cortez C et al. *JAMA Health Forum.*
